# Conceptual approaches in combating health inequity: A scoping review protocol

**DOI:** 10.1371/journal.pone.0282858

**Published:** 2023-03-15

**Authors:** Michelle Amri, Liban Mohamood, Cristián Mansilla, Kathryn Barrett, Jesse B. Bump

**Affiliations:** 1 Takemi Program in International Health, Harvard T.H. Chan School of Public Health, Harvard University, Boston, MA, United States of America; 2 Dalla Lana School of Public Health, Toronto, Ontario, Canada; 3 Department of Health and Society, University of Toronto Scarborough, Toronto, Ontario, Canada; 4 McMaster Health Forum, McMaster University, Hamilton, Canada; 5 Department of Health Research Methods, Evidence and Impact, McMaster University, Hamilton, Canada; 6 University of Toronto Scarborough Library, Scarborough, ON, Canada; 7 Bergen Centre for Ethics and Priority Setting, University of Bergen, Bergen, Norway; Drexel University, UNITED STATES

## Abstract

**Introduction:**

What are the different ways in which health equity can be sought through policy and programs? Although there is a central focus on health equity in global and public health, we recognize that stakeholders can understand health equity as taking different approaches and that there is not a single conceptual approach. However, information on conceptual categories of actions to improve health equity and/or reduce health inequity is scarce. Therefore, this study asks the research question: “what conceptual approaches exist in striving for health equity and/or reducing health inequity?” with the aim of presenting a comprehensive overview of approaches.

**Methods:**

A scoping review will be undertaken following the PRISMA guidelines for Scoping Reviews (PRISMA-ScR) and in consultation with a research librarian. Both the peer-reviewed and grey literatures will be searched using: Ovid MEDLINE, Scopus, PAIS Index (ProQuest), JSTOR, Canadian Public Documents Collection, the World Health Organization IRIS (Institutional Repository for Information Sharing), and supplemented by a Google Advanced Search. Screening will be conducted by two independent reviewers and data will be charted, coded, and narratively synthesized.

**Discussion:**

We anticipate developing a foundational document compiling categories of approaches and discussing the nuances inherent in each conceptualization to promote clarified and united action.

## Introduction

Health equity is a central concept in the fields of public and global health, with organizations like the World Health Organization (WHO) signalling their aim to improve health equity since their inception [1] and solidifying these commitments in subsequent declarations (e.g., the Alma-Ata Declaration [2, 3]). What differentiates a health inequity from an inequality in the public health literature is the social justice aspect, in other words, a moral component entailing a need to act [4]. This builds on a widely cited definition of inequity in health, which is defined as “differences which are *unnecessary* and *avoidable*, but in addition are considered *unfair* and *unjust*” [5]. However, the different ways in which public policy can tackle these unnecessary, avoidable, unfair, and unjust differences is less defined.

A recent scoping review investigating how the peer-reviewed literature understands the WHO’s approach to health equity found that the WHO conceptually approached action in two ways: (i) striving for a baseline level of health and (ii) striving for a baseline level of health and reducing inequality [6, 7]. Drawing on this finding, empirical analysis of key texts of the WHO found that the WHO presents “three main approaches to reduce health inequities” (or “three main approaches to reducing urban health inequities” in *Hidden Cities*) [8, 9]. These three approaches are: “targeting disadvantaged population groups or social classes” (or “targeting disadvantaged groups” in *Hidden Cities*), “narrowing the health gap”, and “reducing inequities throughout the whole population” (or “reducing inequities throughout the entire urban population” in *Hidden Cities*) [8, 9]. Aligned with the WHO’s “three main approaches” discussed above, a briefing note, *Policy Approaches to Reducing Health Inequalities*, produced by the Canadian National Collaborating Centre for Healthy Public Policy, also presents three similar approaches: “focus on disadvantages”, “focus on gaps”, and “focus on gradient” [10]. However, with the slight difference of “focus on gaps” discussing the lowest income group with respect to all other groups, whereas the WHO “narrowing the health gap” focuses on closing the gap between the richest or best off with the poorest or worst off 20% (or quintiles).

In addition to these outlined conceptual approaches, recent analysis demonstrated that the WHO’s discourses also presented four additional ways action could be conceptualized [11]. These approaches were construed through investigating the discourses and language employed in key WHO texts. However, these approaches were not explicitly presented and discussed by the WHO in the way the above three approaches were.

Moving beyond investigations into the WHO, understandings of how health equity can be acted on or sought, or alternatively, how health inequity can be tackled, are limited. For instance, it is commonplace for literature to discuss addressing health inequity as synonymous with targeting one group, as seen by sweeping concluding statements in journal articles to address health equity in this way. This view neglects other approaches, such as those discussed above, which both means that certain populations will be ignored and that different understandings of what health equity entails will not be considered. Failing to consider different conceptual approaches for acting on health equity is particularly problematic in global health, where there is both widespread attention paid to health equity and there are cross-country differences in understandings of social justice and health equity [12]. Further, taking such a narrow view of health equity also tends to lend itself to a focus on methods or technical strategies to improve health equity, such as distributing resources. Although such strategies can be helpful, these actions are often not associated with broader equity considerations, such as reflecting on the unique needs of individuals that may result in different outcomes, given individuals’ differing capabilities [13]. Thus, the focus of this work is to collate information on what conceptual approaches exist, rather than specific methods or strategies which may or may not come through in the discussion of such conceptual approaches. This work is important because a lack of conceptual clarity on the differing approaches to action can prevent united action and potentially inhibit public policy action altogether.

### Objective

The aim of this study is to systematically gather what has been written on the subject and develop a comprehensive understanding of these categories of how health equity can be sought or health inequity can be reduced. Therefore, this scoping review asks the research question: “what conceptual approaches exist in striving for health equity and/or reducing health inequity?” The aim is to develop a list of categorizations with an associated diagram to facilitate understanding.

## Methods

A scoping review will be undertaken to compile English-language hits from numerous sources, including from the peer-reviewed and grey literatures. This scoping review protocol was developed following the Preferred Reporting Items for Systematic Reviews and Meta-Analyses extension for Scoping Reviews (PRISMA-ScR) [14], PRISMA guidelines for protocols (PRISMA-P) [15], the updated methodological guidance for the conduct of scoping reviews [16], and in consultation with a research librarian with expertise in library science.

### Eligibility criteria

#### Inclusion criteria

Texts will be included if they meet the inclusion criteria of providing a conceptual category or categories of approaches in striving for health equity and/or seeking to reduce health inequity, such as those discussed in the introduction. In the instance where texts provide both a discussion of a conceptual category or categories and also include an application (e.g., using data) to illustrate, these will be included. Please note, there are no restrictions on study designs (i.e., commentaries are included). Only texts available in English will be included due to resource constraints.

#### Exclusion criteria

Texts will be excluded if they do not provide a conceptual category or categories in striving for health equity and/or seeking to reduce health inequity. In other words, if a text discusses health equity generally but does not explicitly discuss a specific approach or approaches in striving for health equity, this would be excluded.

#### Information sources and search strategy

The search will be conducted from database inception to date searched in: Ovid MEDLINE, Scopus, PAIS Index (ProQuest), JSTOR, Canadian Public Documents Collection, and the World Health Organization IRIS (Institutional Repository for Information Sharing). This search is further supplemented by a Google Advanced Search to search domains ending in “.org”, “.int”, and “.ca”, with the former two domain endings selected to search intergovernmental and nonprofit organizations’ webpages, and the latter being included due to locating a relevant text and belief that we may find similar documents of relevance under the “.ca” domain ending. Through searching across numerous databases, articles within the medical sciences, public health, public policy, and other disciplines should be retrieved. Similarly, grey literature from both the WHO and others should be pulled through the searches to be conducted in IRIS and Google. Thus, casting a wide net to retrieve articles across numerous disciplines and grey literature from various entities. The proposed database or location of each search, associated rationale, and search strings are provided in S1 File. For example, Scopus will be searched using the following search string: (TITLE-ABS-KEY ("health equit*" OR "health inequit*" OR "health equalit*" OR "health inequalit*") ) AND (TITLE-ABS-KEY ("public polic*" OR "health polic*" OR "social polic*") ) AND (LIMIT-TO (DOCTYPE, "ar") ) AND (LIMIT-TO (LANGUAGE, "English") ).

### Study records

#### Data management

Extracted hits will be imported into Covidence [17] software, where duplicates will be removed. Given that this is a comprehensive search, the search will span across both the peer-reviewed and grey literatures.

#### Selection process

The first stage of screening will entail two independent reviewers assessing both the title and abstract for peer-reviewed articles. For grey literature hits where no abstract appears, the table of contents and executive summary will be reviewed. After the first stage of screening is complete, conflicts will be resolved by a third reviewer. Following the first screen, articles marked for full text review will be read by two independent reviewers to ensure alignment with the inclusion criteria (and not falling into the exclusion criteria). Any discrepancies will be reviewed by one additional member of the team, who will accordingly determine the final articles included in the study.

#### Data collection process and data extraction

A data extraction template will be used to compile information on categories of conceptualizations that appear in included papers and the bibliographic information of author(s), publication year, and publication title. Although we anticipate compiling more conceptual categories beyond those presented by the WHO and the Canadian National Collaborating Centre for Healthy Public Policy, we do not anticipate charting additional categories of data but remain open to including any additional relevant information, similar to the approach taken in other studies [18] and recommended in the updated guidance for undertaking scoping reviews [16]. The development of the data extraction template will be led by one author and piloted using an initial set of included papers (~n = 10) and reviewed by two independent reviewers, as has also been done in other studies [19, 20]. And similarly, once the categories of conceptualizations are agreed upon, NVivo [21] software will be used to undertake coding both deductively using the a priori codes of the “three main approaches” discussed above, and inductively, through compilation of additional conceptual approaches. Data will be subsequently narratively synthesized to form a comprehensive list for understanding how health inequities can be tackled and an associated diagram will be produced to facilitate understanding. All authors will review both the charted and extracted data carefully.

#### Limitations

Because only English papers are included in this study due to resource constraints, this poses a limitation for ensuring no hits are missed.

## Discussion

Despite health equity being mainstream and commonplace in the fields of global and public health, discussions around health equity tend to remain vague, as noted in a critical discourse analysis of key WHO texts [22]. This lack of clarity also translates into policy and practice. This was observed in a recent study of key informants with experience with the WHO’s Urban Health Equity Assessment and Response Tool (Urban HEART) [12]. Urban HEART is a tool for cities to address health inequities [23] and has been designed to be comprehensive, inclusive, operationally feasible, among other things [24, 25]. Despite key informants accepting health equity as a central concept, they defined the concept in different ways and found the concept to be vague [12]. This study demonstrates that the concept of health equity can be unclear even to individuals whose work has focused on health equity.

Therefore, we anticipate discussing the nuances inherent in each conceptualization and developing a foundational document to promote clarified and united action. This study has widespread relevance for work at various levels, including: population-level efforts that are directly and indirectly focused on improving health equity, such as the Health in All Policies approach and Healthy Cities [26]; individual-level efforts that seek to improve health equity, such as clinicians working on poverty screening [27]; organizational efforts, particularly in light of the persistence of inequities in global health institutions [28]; and others. This work is not only unique, it is particularly timely given COVID-19, where there has been a call for focusing on achieving equitable outcomes instead of cutting spending [29] and observations that the pandemic may afford an opportunity to refocus on the social determinants of health and health equity [30].

## Supporting information

S1 File (PDF)

## List of abbreviations

PRISMA-P: Preferred Reporting Items for Systematic review and Meta-Analysis Protocols
PRISMA-ScR: Preferred Reporting Items for Systematic Reviews and Meta-Analyses extension for Scoping Reviews (PRISMA-ScR
Urban HEART: Urban Health Equity Assessment and Response Tool
WHO: World Health Organization
