# Supplementary material for: Conceptual approaches in combating health inequity: A scoping review protocol
Source: PLoS One. 2023 Mar 15;18(3):e0282858. doi: 10.1371/journal.pone.0282858 (PMC10016682; doi:10.1371/journal.pone.0282858)
Supplement: S1 File — (PDF) [file pone.0282858.s001.pdf]

Table 1 *Location to be searched, associated rationale, and search strings*

**Database or location**	**Rationale**	**Search string or approach**
Ovid MEDLINE	Ovid MEDLINE includes biomedical and life science journals.	**# — Search Statement**<br>1 Health Equity/<br>2 ((health adj1 equit$) or (health adj1 inequit$) or (health adj1 equalit$) or (health adj1 inequalit$)).tw,kf.<br>3 1 or 2<br>4 public policy/ or exp health policy/<br>5 ((public adj1 polic$) or (health adj1 polic$) or (social adj1 polic$)).tw,kf.<br>6 4 or 5<br>7 3 and 6<br>8 limit 7 to journal article<br>9 limit 8 to english language
Scopus	Scopus is multidisciplinary in nature and includes the fields of medicine and social sciences.	(TITLE-ABS-KEY ("health equit*" OR "health inequit*" OR "health equalit*" OR "health inequalit*")) AND (TITLE-ABS-KEY ("public polic*" OR "health polic*" OR "social polic*")) AND (LIMIT-TO (DOCTYPE , "ar")) AND (LIMIT-TO (LANGUAGE , "English"))

PAIS Index (ProQuest)	The PAIS Index includes international sources and includes a range of public policy materials, including articles, grey literature, research reports, and web content.	((MAINSUBJECT.EXACT.EXPLODE("Health") AND (MAINSUBJECT.EXACT("Equality") OR MAINSUBJECT.EXACT.EXPLODE("Inequality") OR MAINSUBJECT.EXACT("Equity"))) OR noft("health equit*" OR "health inequit*" OR "health equalit*" OR "health inequalit*")) AND ((MAINSUBJECT.EXACT("Social Policy") OR MAINSUBJECT.EXACT("Public Policy") OR MAINSUBJECT.EXACT("Health Policy")) OR noft("public polic*" OR "social polic*" OR "health polic*")) AND (stype.exact("Scholarly Journals") AND la.exact("ENG") AND PEER(yes))
JSTOR	JSTOR contains academic journals, books, and primary sources, and was selected due to being more of a curated collection that provides results from a selection of hand-picked, full-text, archived journals.	("health equity" OR "health equities" OR "health inequity" OR "health inequities" OR "health equality" OR "health equalities" OR "health inequality" OR "health inequalities") [all fields] AND (polic~) [all fields]<br>Narrow results: Articles [item type], English [language]<br>All content (not just content we can access)<br><br>The JSTOR search was adapted because it was not possible to search for truncated terms within phrases, and because search strings could only be 200 characters or less. “All fields” was selected because other options were narrower than existing searches, so we selected this to be more comprehensive.
Canadian Public Documents Collection	The Canadian Public Documents Collection is focused on Canadian public policy and contains	“health equity” [anywhere] AND policy [anywhere]

	publications from government and non-governmental organizations.	
World Health Organization IRIS (Institutional Repository for Information Sharing)	IRIS is a grey literature repository of WHO texts that was selected because the WHO is a key health agency.	All of IRIS: “health equity”<br>Limits: Language equals “en” AND Document type equals Publications
Google Advanced Search	Searching Google will allow for the compilation of grey literature from a wide range of sources, namely intergovernmental and nonprofit organizations and Canadian webpages, that are unlikely to be retrieved from the above searches.	First ten pages to be searched for the following three search strings:<br>policy health equity OR inequity OR equality OR inequality site:.org filetype:pdf<br>policy health equity OR inequity OR equality OR inequality site:.ca filetype:pdf<br>policy health equity OR inequity OR equality OR inequality site:.int filetype:pdf

Preferred Reporting Items for Systematic reviews and Meta-Analyses extension for Scoping Reviews (PRISMA-ScR) Checklist

SECTION	ITEM	PRISMA-ScR CHECKLIST ITEM	REPORTED ON PAGE #
**TITLE**			
Title	1	Identify the report as a scoping review.	Title (page 1)
**ABSTRACT**			
Structured summary	2	Provide a structured summary that includes (as applicable): background, objectives, eligibility criteria, sources of evidence, charting methods, results, and conclusions that relate to the review questions and objectives.	Abstract (page 2)
**INTRODUCTION**			
Rationale	3	Describe the rationale for the review in the context of what is already known. Explain why the review questions/objectives lend themselves to a scoping review approach.	3
Objectives	4	Provide an explicit statement of the questions and objectives being addressed with reference to their key elements (e.g., population or participants, concepts, and context) or other relevant key elements used to conceptualize the review questions and/or objectives.	4
**METHODS**			
Protocol and registration	5	Indicate whether a review protocol exists; state if and where it can be accessed (e.g., a Web address); and if available, provide registration information, including the registration number.	This is the protocol
Eligibility criteria	6	Specify characteristics of the sources of evidence used as eligibility criteria (e.g., years considered, language, and publication status), and provide a rationale.	4
Information sources*	7	Describe all information sources in the search (e.g., databases with dates of coverage and contact with authors to identify additional sources), as well as the date the most recent search was executed.	4-5
Search	8	Present the full electronic search strategy for at least 1 database, including any limits used, such that it could be repeated.	6-8
Selection of sources of evidence†	9	State the process for selecting sources of evidence (i.e., screening and eligibility) included in the scoping review.	9
Data charting process‡	10	Describe the methods of charting data from the included sources of evidence (e.g., calibrated forms or forms that have been tested by the team before their use, and whether data charting was done independently or in duplicate) and any processes for obtaining and confirming data from investigators.	9
Data items	11	List and define all variables for which data were sought and any assumptions and simplifications made.	9
Critical appraisal of individual sources of evidence§	12	If done, provide a rationale for conducting a critical appraisal of included sources of evidence; describe the methods used and how this information was used in any data synthesis (if appropriate).	N/A

St. Michael's
Inspired Care.
Inspiring Science.

SECTION	ITEM	PRISMA-ScR CHECKLIST ITEM	REPORTED ON PAGE #
Synthesis of results	13	Describe the methods of handling and summarizing the data that were charted.	9
**RESULTS**			
Selection of sources of evidence	14	Give numbers of sources of evidence screened, assessed for eligibility, and included in the review, with reasons for exclusions at each stage, ideally using a flow diagram.	To be done in full manuscript
Characteristics of sources of evidence	15	For each source of evidence, present characteristics for which data were charted and provide the citations.	To be done in full manuscript
Critical appraisal within sources of evidence	16	If done, present data on critical appraisal of included sources of evidence (see item 12).	N/A
Results of individual sources of evidence	17	For each included source of evidence, present the relevant data that were charted that relate to the review questions and objectives.	To be done in full manuscript
Synthesis of results	18	Summarize and/or present the charting results as they relate to the review questions and objectives.	To be done in full manuscript
**DISCUSSION**			
Summary of evidence	19	Summarize the main results (including an overview of concepts, themes, and types of evidence available), link to the review questions and objectives, and consider the relevance to key groups.	To be done in full manuscript
Limitations	20	Discuss the limitations of the scoping review process.	9
Conclusions	21	Provide a general interpretation of the results with respect to the review questions and objectives, as well as potential implications and/or next steps.	To be done in full manuscript
**FUNDING**			
Funding	22	Describe sources of funding for the included sources of evidence, as well as sources of funding for the scoping review. Describe the role of the funders of the scoping review.	11

JBI = Joanna Briggs Institute; PRISMA-ScR = Preferred Reporting Items for Systematic reviews and Meta-Analyses extension for Scoping Reviews.
* Where *sources of evidence* (see second footnote) are compiled from, such as bibliographic databases, social media platforms, and Web sites.
† A more inclusive/heterogeneous term used to account for the different types of evidence or data sources (e.g., quantitative and/or qualitative research, expert opinion, and policy documents) that may be eligible in a scoping review as opposed to only studies. This is not to be confused with *information sources* (see first footnote).
‡ The frameworks by Arksey and O'Malley (6) and Levac and colleagues (7) and the JBI guidance (4, 5) refer to the process of data extraction in a scoping review as data charting.
§ The process of systematically examining research evidence to assess its validity, results, and relevance before using it to inform a decision. This term is used for items 12 and 19 instead of "risk of bias" (which is more applicable to systematic reviews of interventions) to include and acknowledge the various sources of evidence that may be used in a scoping review (e.g., quantitative and/or qualitative research, expert opinion, and policy document).

From: Tricco AC, Lillie E, Zarin W, O'Brien KK, Colquhoun H, Levac D, et al. PRISMA Extension for Scoping Reviews (PRISMAScR): Checklist and Explanation. Ann Intern Med. 2018;169:467–473. doi: 10.7326/M18-0850.
